# Impact of Ultra-High-Pressure Homogenization of Buttermilk for the Production of Yogurt

**DOI:** 10.3390/foods10081757

**Published:** 2021-07-29

**Authors:** Louise Krebs, Amélie Bérubé, Jean Iung, Alice Marciniak, Sylvie L. Turgeon, Guillaume Brisson

**Affiliations:** 1 STELA Dairy Research Centre, Department of Food Sciences, Institute of Nutrition and Functional Foods (INAF), Université Laval, Québec, QC G1V 0A6, Canada; louise.krebs.1@ulaval.ca (L.K.); amelie.berube.3@ulaval.ca (A.B.); jean.iung.1@ulaval.ca (J.I.); alice.marciniak.1@ulaval.ca (A.M.); sylvie.turgeon@fsaa.ulaval.ca (S.L.T.); 2 Faculty of Health, Medicine and Life Sciences, Maastricht University, 6211 LK Maastricht, The Netherlands

**Keywords:** buttermilk, yogurt, MFGM, ultra-high-pressure homogenization

## Abstract

Despite its nutritional properties, buttermilk (BM) is still poorly valorized due to its high phospholipid (PL) concentration, impairing its techno-functional performance in dairy products. Therefore, the objective of this study was to investigate the impact of ultra-high-pressure homogenization (UHPH) on the techno-functional properties of BM in set and stirred yogurts. BM and skimmed milk (SM) were pretreated by conventional homogenization (15 MPa), high-pressure homogenization (HPH) (150 MPa), and UHPH (300 MPa) prior to yogurt production. Polyacrylamide gel electrophoresis (PAGE) analysis showed that UHPH promoted the formation of large covalently linked aggregates in BM. A more particulate gel microstructure was observed for set SM, while BM gels were finer and more homogeneous. These differences affected the water holding capacity (WHC), which was higher for BM, while a decrease in WHC was observed for SM yogurts with an increase in homogenization pressure. In stirred yogurts, the apparent viscosity was significantly higher for SM, and the pretreatment of BM with UHPH further reduced its viscosity. Overall, our results showed that UHPH could be used for modulating BM and SM yogurt texture properties. The use of UHPH on BM has great potential for lower-viscosity dairy applications (e.g., ready-to-drink yogurts) to deliver its health-promoting properties.

## 1. Introduction

Buttermilk (BM), which is the serum phase generated during the production of butter, is produced in approximately equal parts as butter [1]. The Canadian dairy industry produced 118,235 metric tons of butter in 2020 [2], leading to an estimated equal volume of BM, while global BM production has been estimated at about 3.2 million tons per annum [3]. Despite the large quantities produced every year, BM is still undervalued. Indeed, BM is mainly used in animal feeds, for its emulsifying capacity or for providing a milk flavor in various food applications [4,5]. As with skim milk (SM), BM is composed of the main milk solids-not-fat, namely caseins (CNs), whey proteins (WPs), lactose, and minerals [6]. The main difference between SM and BM is the phospholipid (PL) content, which, in BM, is up to ten times higher than in SM [7] and four times higher than in raw milk [8]. PLs are part of a very specific structure found in dairy products, the milk fat globule membrane (MFGM) [9], which is released into BM during the churning of cream into butter. The unique composition of PLs in MFGM (phosphatidylcholine-PC, phosphatidylethanolamine-PE, phosphatidylserine-PS, phosphatidylinositol-PI, and sphingomyelin-SM), along with the MFGM proteins, is responsible for its various health benefits [10,11,12]. For example, daily dietary supplementation with BM led to a decrease in concentration of total and LDL cholesterol in moderately hypercholesterolemic men and women [10]. Furthermore, sphingomyelin-fortified milk was found to have a positive influence on infants’ gut microbiota and neurocognitive development [13].

Despite its nutritional and biological properties, compared to SM, BM possesses limited technological properties in dairy applications due to its high PL content [4,14,15]. Thus, the incorporation of BM into dairy matrices, such as yogurt, presents different technological challenges, such as a decrease in firmness [16] and lower apparent viscosity [17], which impacts the quality of the final product. Nevertheless, the incorporation of very small amounts of BM or BM powder (BMP) into yogurt matrices produced interesting results, with a reported decrease in whey separation and syneresis [18], and an increase in water-holding capacity (WHC) [16]. Contradictory impacts on firmness have been reported, which could be due to the various sources and quantities of BM used [16,19]. Thus, the use of mechanical treatment to improve BM’s functional properties and facilitate its incorporation into various dairy matrices is of high interest in order to provide the benefits of its health-promoting activities. In yogurt production, conventional homogenization using pressures between 20 and 60 MPa [20] has already been shown to affect yogurt properties. As a matter of fact, it stabilizes emulsions and decreases the creaming phenomenon of milk [21] by reducing the size of fat globules and simultaneously increasing their surface area [22]. This leads to lipids being denser and more homogeneously dispersed throughout the liquid, resulting in an improved viscosity and WHC of yogurt [23]. The fat globules from heated homogenized milk are known to interact with the proteins in the acid gel network as active filler particles [24]. However, Ji et al. (2011) reported that the extent of interaction of the fat globules with the protein network depends on their size and, therefore, the homogenization pressure [25]. They showed that milk recombined at low homogenization pressures resulted in larger fat globules with less active interaction with the network, while milk treated at higher pressures resulted in smaller globules more tightly bound to the protein network. Similarly, high-pressure homogenization (HPH) (between 150 and 200 MPa) produced even smaller fat globules with increased surface area [20] and, thus, improved properties within the yogurt matrix. A few studies have also reported that the size of homogenized fat globules affects their incorporation in the protein network, especially at higher homogenization pressures and when using microfluidization [25,26,27]. Recently, with the development of high-pressure intensifiers and valve materials (stainless steel, ceramic, and seals) resistant to extremely high homogenization, pressures up to 400 MPa can be reached [20,28,29]. This process is known as ultra-high-pressure homogenization (UHPH), which ranges from 200 to 400 MPa. Applied to whole milk, it causes fat globule reduction to submicron sizes. However, above that pressure, fat globules form clusters through the aggregation of CN and WP at their surface [30], which induces higher WHC and changes in the gel firmness [23,31]. In addition, some authors have reported decreases in CN micelle sizes from 5% to 33% in the UHPH range [21,32,33]. 

Given the interesting results from the application of UHPH for yogurt production from SM, as well as the high nutritional quality of BM, there is much interest in the use of UHPH for the production of BM yogurt. Hence, the aim of this study was to examine the impact of UHPH as a pre-treatment for the production of yogurts from BM compared to those produced from SM. In this paper, set and stirred yogurts were produced and characterized using different homogenization treatments (15, 150, and 300 MPa). This new approach of using UHPH for improving BM use in yogurt is of great interest for BM valorization through the production of a potentially highly functional product enriched in MFGM health-promoting components.

## 2. Materials and Methods

### 2.1. Materials

Whole raw milk and raw cream were provided by a local supplier (Quebec City, QC, Canada) and skim milk powder (SMP) was obtained from Agropur (Quebec City, QC, Canada). The thermophilic yogurt culture YC-X11 Yo-Flex^®^ (Chr. Hansen A/S, Hørsholm, Denmark) was composed of *Streptococcus thermophilus* and *Lactobacillus delbrueckii* subsp. *Bulgaricus*. Analytical-grade sodium hydroxide for the preparation of 0.1 M of NaOH was obtained from Fisher Chemical (Ottawa, ON, Canada). Mini-PROTEAN TGX Stain-Free Gels (12%, 15-well comb, 15 µL), 2× Laemmli sample buffer, native sample buffer, Precision Plus ProteinTM All Blue Standards, 10× Tris/glycine/sodium dodecyl sulfate (SDS) buffer, and 10× Tris/glycine buffer were all obtained from BioRad (Hercules, CA, USA). 2-Mercaptoethanol was provided by Sigma-Aldrich (St. Louis, MO, USA). Methanol was obtained from Fisher Chemical (Ottawa, ON, Canada) and glacial acetic acid from Anachemia (Radnor, PA, USA). Fast Green FCF and Nile Red were obtained from Sigma-Aldrich (Oakville, ON, Canada).

### 2.2. Production of BM and SM Yogurts

**Figure 1:**
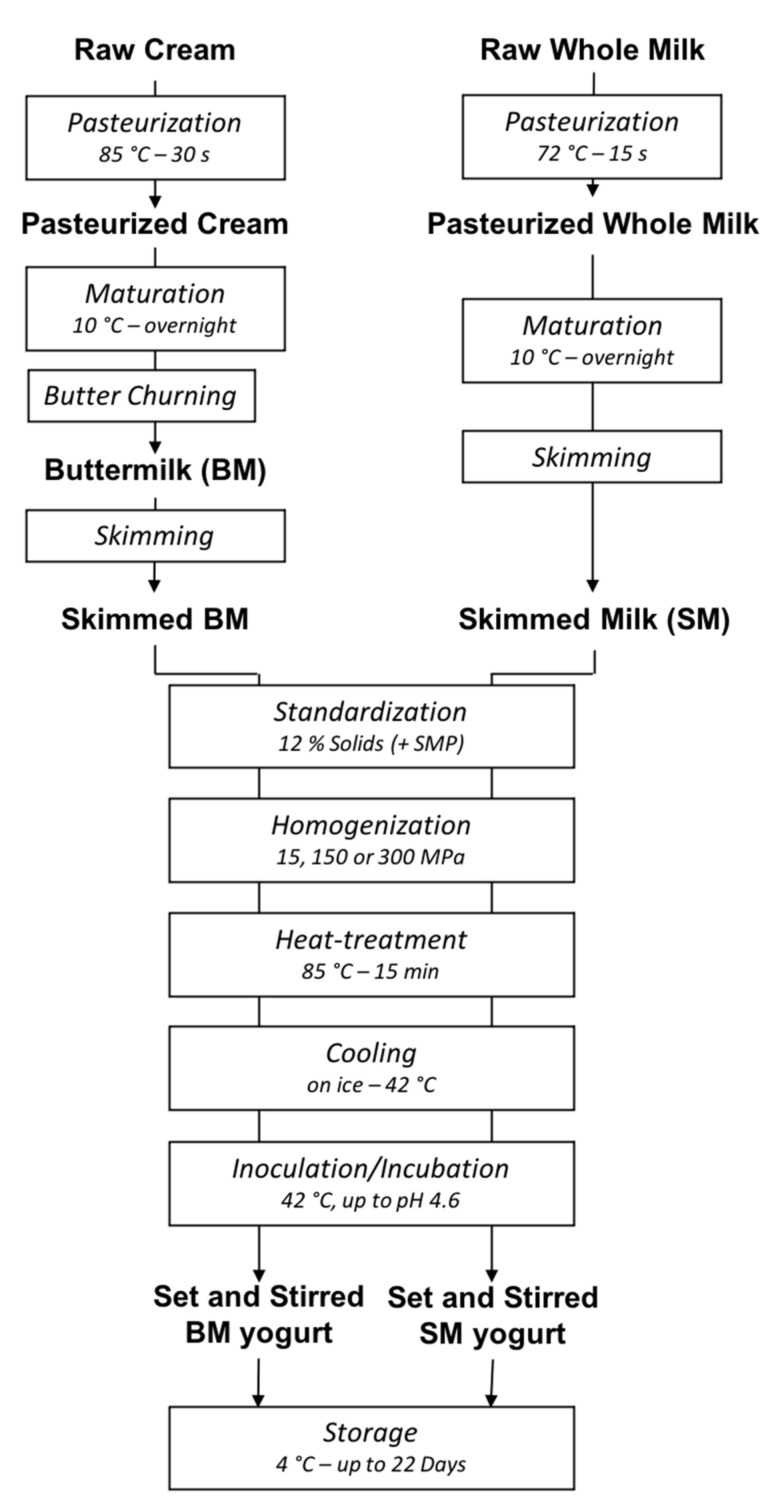
Experimental design of the production of set and stirred yogurts from buttermilk (BM) and skimmed milk (SM) treated by ultra-high-pressure homogenization (UHPH).

The production steps of BM yogurts and SM yogurts (control) are presented in Figure 1. Briefly, raw cream and whole raw milk were pasteurized (Chalinox/Hydro-Québec CFI-25, QC, Canada) at 85 °C for 30 s and 72 °C for 15 s, respectively. Both pasteurized cream and pasteurized milk were matured overnight at 10 °C. Then, to obtain BM, the matured pasteurized cream was churned at 75 rpm at approximately 12 °C using a pilot plant-scale butter churn with a capacity of 8–15 L (Qualtech Equipment, QC, Canada). The pasteurized BM and whole milk were heated for 10 min to 40 °C and cream was separated using a cream separator (Westfalia LWA-205-DeLaval, Lund, Sweden). Skimmed BM and SM (approximately 10% total solids) were enriched with 30 g of SMP per liter, to reach a solid content of approximately 12% (±0.25%) and achieve a similar yogurt firmness as commercial yogurts. The final composition of BM and SM (Table 1) was determined with a LactoScope FTIR milk analyzer (Delta Instruments, Drachten, The Netherlands). Pasteurized BM and SM were aliquoted into 3 batches of 2 L. Each batch was used for one homogenization parameter of 15 MPa, 150 MPa, and 300 MPa using a UHPH system (Nano Debee Model 45-4, Bee International, South Easton, MA, USA). 

After UHPH treatment, BM and SM were heated at 85 °C on a stove for 15 min (temperature rise time of approximately 10 min), with continuous stirring, and then cooled on ice to 42 °C. Starter cultures (YC-X11 Yo-Flex^®^) were added according to the manufacturer’s directions [34]. Briefly, 0.05 g of the frozen culture was added to 2 L of treated BM or SM, stirred for several minutes until complete dissolution, and subsampled for further analysis. All samples were incubated at 42 °C to reach a pH of 4.6 (approximately 6 h for BM yogurts and 8 h for SM yogurts) and were then stored at 4 °C overnight. For set yogurt, incubation took place in 100 mL plastic containers and 50 mL tubes, and yogurts were stored at 4 °C until further analysis. Stirred yogurts were produced by manual stirring with a metal spoon (30 times clockwise and 30 times anticlockwise) to break down the gel. The six stirred yogurts (3 pressure levels-15, 150, and 300 MPa, and 2 sources-BM and SM) were stored at 4 °C until further analysis.

### 2.3. Protein Profiles of Homogenized BM and SM

The protein profiles of all fluid BM and SM samples after homogenization treatment were determined by native polyacrylamide gel electrophoresis (PAGE) and SDS-PAGE under nonreducing conditions. In addition, SM and BM controls were prepared with samples treated at 15 MPa, under reducing conditions (50 µL of 2-mercaptoethanol and 950 µL of Laemmli buffer). All samples were diluted in distilled water (1:9), and 25 µL of each dilution was mixed with 25 µL of their respective sample buffer. Solutions were then loaded onto precast 12% acrylamide gels in a Mini PROTEAN^®^ Tetra Cell (BioRad, Hercules, CA, USA). Precision Plus Protein™ All Blue Standards with molecular weights ranging from 10 to 250 kDa were used as the molecular weight marker. The electrophoresis was conducted at a constant voltage of 120 V for 1 h. The gels were then stained with Coomassie blue solution (BioRad, Hercules, CA, USA) for 1 h, followed by de-staining with a mixture of methanol, acetic acid, and distilled water (1:1:8) overnight. The gels were scanned the next day using a ChemiDoc™ MP imaging system (Bio-Rad, Hercules, CA, USA).

### 2.4. Physico-Chemical Characteristics of Set and Stirred Yogurts

Physico-chemical characterization of set and stirred yogurts was performed using the following analysis and was repeated after storage at 4 °C on days 1, 8, 15, and 22 after yogurt production.

#### 2.4.1. pH

The pH of set and stirred yogurts was measured using a pH meter (Orion Star T910, Thermo Fisher Scientific, Waltham, MA, USA) calibrated with standardized buffer solutions (pH 4.0 and 7.0).

#### 2.4.2. Firmness

The firmness of set yogurts was assessed according to Le et al. (2011) with slight modifications [16]. A penetration test was carried out on set yogurts at 4 °C using a texturometer (TA.XT2, Texture Technologies, New York, NY, USA). A 25 mm-diameter cylindrical probe was used at a constant rate of 1 mm/s for a distance of 40 mm. The maximum force recorded in real-time represents the firmness (N).

#### 2.4.3. Water-Holding Capacity

The WHC of set yogurts was measured on days 1, 8, 15, and 22 after yogurt production according to Le et al. (2011) [16]. Samples were centrifuged (IEC Centra CL2 centrifuge, Thermo Fisher Scientific Inc., Milford, MA, USA) at 1200× *g* for 15 min at 4 °C. The top layer (whey) was removed and weighed, and the WHC was calculated according to the following equation.

(1) $$\mathrm{WHC}\;\left(\%\right)=\left[\frac{\left(\mathrm{sample}\;\mathrm{weight}\;\left(g\right)-\mathrm{expelled}\;\mathrm{whey}\left(g\right)\right)}{\mathrm{sample}\;\mathrm{weight}\;\left(g\right)}\right]\times 100$$

#### 2.4.4. Confocal Laser Scanning Microscopy

The microstructure of set and stirred yogurts was investigated using confocal laser scanning microscopy (CLSM) based on the methods of Lucey et al. (1998) and Zhao et al. (2016) with some adaptations [24,35]. Following milk inoculation, milks were incubated at 42 °C for 1 h. A volume of 960 µL of BM or SM was then mixed with 20 µL of 1% Fast Green (proteins) and 20 µL of 2% Nile Red (PLs). After a 15 min waiting period with intermittent stirring, 3 mL of BM or SM was added and thoroughly mixed. Mixtures were poured into a 35 mm culture dish with a 15 mm glass bottom and placed in the incubator for fermentation (approximately 9 h). Stirred yogurts were produced by manual stirring with a metal spoon. Imaging was performed using a STELLARIS5 confocal microscope (LEICA, Mannheim, Germany) equipped with an HC PL APO CS 40×/0.853 NA air objective. The sample was mounted in a MatTek coverslip bottom 35 mm culture dish with a 14 mm glass diameter (MatTek life science, Ashland, MA, USA) and excited with lasers at 552 nm and 638 nm, separately, for Nile red and Fast Green FCF, respectively. The emission was acquired with a variable dichroic selecting wavelength over 540–600 nm for Nile red and over 640–800 nm for Fast Green FCF.

#### 2.4.5. Titratable Acidity

The titratable acidity of stirred yogurt was determined at 20 °C according to the AOAC method 947.05 [36]. Samples were prepared by mixing 10 g of yogurt in 40 mL of distilled water. Samples were continuously stirred and titrated using 0.1 M of NaOH to a pH of 8.3 using an automatic titrator (Orion Start T910, Thermo Fisher Scientific, Waltham, MA, USA). The amount of titrant needed to reach pH 8.3 was noted, and the titratable acidity (% lactic acid) was calculated according to the following equation.

(2) $$Titrable\;acidity\;\left(\%\;lactic\;acid\right)=\frac{\mathrm{titrant}\;\left(\mathrm{mL}\right)}{\mathrm{sample}\;\mathrm{weight}\;\left(g\right)}\times 0.9$$

#### 2.4.6. Apparent Viscosity

A temperature-controlled rheometer (TA instruments, model ARES-G2, New Castle, DE, USA) was used for the measurements of apparent viscosity of stirred yogurts at 10 °C, according to Yu, Wang, and McCarthy (2016) with some adaptations [37]. The measuring system consisted of a 40 mm, 0.04 radius cone and plate geometry. Shear rate sweep (1 to 120 s^−1^) and shear stress response tests were performed. Apparent viscosity was measured at a shear rate of 50 s^−1^.

#### 2.4.7. Drained Syneresis

Drained syneresis of the stirred yogurts was measured at 4 °C, according to Hassan et al. (1996) with some modifications [38]. A mesh screen (Cell strainer, pluriSelect USA, El Cajon, CA, USA) with a mesh size of 200 µm and a radius of 20 mm was used, and mesh tension was released by running water through the mesh. The yogurt sample (4 g) was poured on a mesh screen placed above a previously weighed empty centrifuge tube. Samples were left for 2 h at 4 °C in order to collect the whey resulting from the syneresis. The weight of the centrifuge tubes was measured after sample draining, and the drained syneresis was calculated according to the following equation.

(3) $$Drained\;synerisis=\frac{drained\;whey\;\left(g\right)}{sample\;weight\;\left(g\right)}\times 100$$

### 2.5. Statistical Analysis

Four independent productions of yogurts (replicates) were performed with different batches of raw cream and whole raw milk. Analyses were conducted for each replicate. Data were processed using a multi-factor ANOVA and SAS software (SAS University Edition) to compare BM and SM yogurt properties. Significant differences between pressure treatments and storage time were evaluated with the Tukey test. Evaluations were based on a significance level of *p* < 0.05.

## 3. Results and Discussion

### 3.1. Impact of UHPH Treatment on Protein Profiles of BM and SM

**Figure 2:**
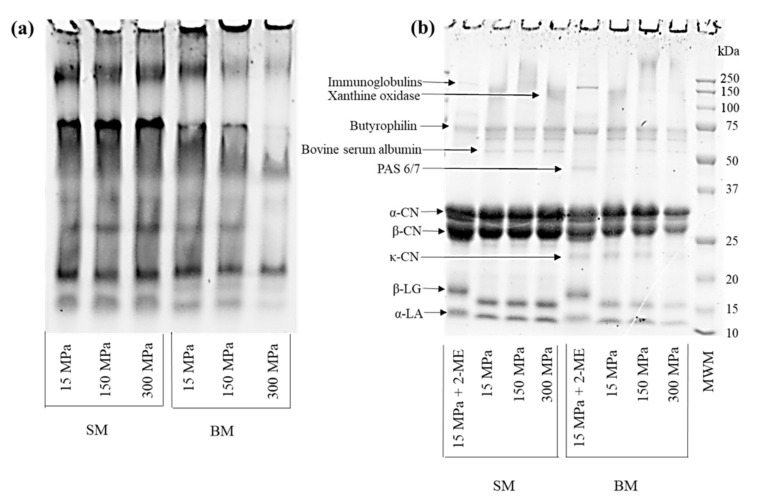
Acrylamide gels (12%) of skimmed milk (SM) and buttermilk (BM) following pressure treatments ((**a**) native polyacrylamide gel electrophoresis (PAGE) pattern, and (**b**) sodium dodecyl sulfate (SDS)-PAGE pattern under reducing (lanes 1 and 5) and nonreducing conditions). PAS 6/7 = periodic acid Schiff 6/7, MWM = molecular weight markers, 2-ME = 2-mercaptoethanol.

To study the impact of UHPH on BM and SM proteins, their profiles were analyzed using native PAGE and nonreducing SDS-PAGE (Figure 2a,b). Different profiles were observed for BM and SM samples for native PAGE (Figure 2a). UHPH treatment did not have as large an impact on SM as it did on BM. Similar band patterns were observed for SM between all the homogenization pressures tested, but more drastic changes were observed for the BM samples. Indeed, an overall decrease was observed in the intensity of the BM protein bands migrating within the gel as the homogenization pressure increased from 150 MPa to 300 MPa compared to the BM treated with a conventional homogenization pressure (15 MPa). This decrease indicates that the main milk proteins underwent denaturation and aggregation upon UHPH, the severity of which was dependent on the pressure used. Concomitantly, a proportional increase in the intensities of the protein signals in the loading wells was observed for the BM samples homogenized at 150 MPa and 300 MPa, confirming that large protein aggregates formed during higher homogenization treatments. The BM and SM samples were then analyzed by SDS-PAGE (Figure 2b) under nonreducing (lanes 2−4 for SM and 6−8 for BM) and reducing conditions (lane 1 for SM and lane 5 for BM) to determine the nature of these interactions. UHPH treatment of SM (lanes 2–4) did not show any effects among the pressures tested (150 MPa and 300 MPa), as can be seen from their similar profiles. The SM protein band intensities were similar for all pressure treatments and were comparable to the samples under reducing conditions (lane 1), indicating that a very low polymerization and aggregation of milk proteins occurred in SM during UHPH. However, gel electrophoresis demonstrated an important impact of the UHPH treatment on the protein aggregation in BM. First, bands corresponding to WP (α-lactalbumin (α-LA) and β-lactoglobulin (β-LG)) decreased in intensity as the homogenization pressure increased. This result is in agreement with Lopez-Fandiño, Carrascosa, and Olano (1996), who observed increasing denaturation and aggregation of WP, more precisely β-LG, at pressures from 100 to 400 MPa [39]. A possible explanation for the lower impact of UHPH on SM protein aggregation could be that SM underwent lower pasteurization temperatures (72 °C/15 s) than those of the cream used for the production of BM (85 °C/30 s). It is known that the pasteurization of cream induces important WP denaturation and the formation of aggregates, especially for β-LG, through intermolecular disulfide interactions with the CN and the MFGM proteins, the extent of which depends on the severity of the thermal treatment [40]. Finally, bands corresponding to MFGM proteins (visible on lane 5) are not detected in UHPH-treated BM samples (lanes 6−8). This suggests strong covalent interactions between WPs, especially β-LG, CN micelles, and MFGM proteins, which are also attributable to the increase in temperature during pressure treatment [41]. These results indicate that more protein denaturation takes place in BM than in SM due to BM’s higher MFGM content, and, as mentioned above, due to the higher temperature applied during the pasteurization of cream in the production of BM. Thus, the more severe pasteurization treatment for cream leads to more potential interactions between MFGM fragments, notably through the MFGM proteins and WPs under thermal treatment of cream, as observed by Morin et al. (2007) [42].

### 3.2. Impact of UHPH Treatment on Physico-Chemical Properties of Set BM and SM Yogurt

**Figure 3:**
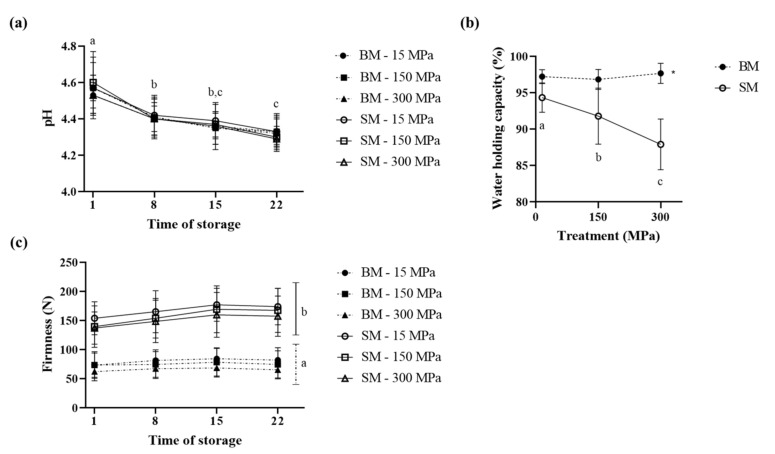
Evolution of physico-chemical properties ((**a**) pH, (**b**) water-holding capacity-WHC, and (**c**) firmness) for set buttermilk (BM-stippled line) and skimmed milk (SM-plain line) yogurts as a function of time of storage (1, 8, 15, and 22 days) and pressure levels (15, 150, and 300 MPa).

Figure 3 presents the physico-chemical and textural properties ((a) pH, (b) WHC, and (c) firmness) of set yogurt as a function of dairy source (SM: plain line, and BM: stippled line), pressure treatment (15, 150, and 300 MPa), and storage time (days 1, 8, 15, and 22). The pH of set yogurt was not influenced by the dairy source or homogenization pressure applied (Figure 3a). However, a significant decrease in pH was noticed during the storage time. After storage at 4 °C, and regardless of the dairy source and pressure treatment, a significant drop in pH was observed from 4.58 at day 1 to 4.42 at day 8, finally reaching 4.33 at day 22. This expected effect is attributed to fermentation of residual lactose by the starter cultures [43].

The WHC of set yogurt indicates the ability of the yogurt gel structure to retain water [44]. A low value for WHC is associated with an unstable yogurt gel network [45]. As observed in Figure 3b, the dairy source and pressure treatment applied highly influence the WHC of set yogurt in an interactive way. BM yogurts had a higher WHC than SM yogurts did regardless of the storage time and treatment, with an average of 97.26% for BM in contrast to 92.14% for SM. These results agreed with the study of Le et al. (2011). These authors observed increases in WHC of 7, 15, 21, and 31%, when they replaced 1, 2, 3, and 4% of the original total SM solids in their yogurt mix with equivalent amounts of solids from a MFGM isolate, whereas substituting SM with BMP at the same ratios did not impact the WHC [16]. However, other authors reported an increase in WHC when low-fat yogurts (12% total solids) were enriched with 1% and 2% of BMP [19]. In our study, 8% of the total 12% solids originated from BM solids. We also calculated that MFGM represented approximately 0.37% of the 12% total solids of our yogurt mix (based on an average MFGM extraction yield of 3.5 g of MFGM/L of BM using a common method [46], results not shown). Overall, the higher WHC of BM yogurt can be explained by its composition, more precisely, the PL content, which is higher in BM than in SM. Indeed, PLs show amphiphilic characteristics, allowing increased retention of water [16,47], while simultaneously, milk proteins have excellent WHCs [48]. Interactions between PLs and WPs or β-CN via electrostatic and hydrophobic connections (Gallier et al., 2012), as well as interactions between MFGM proteins and CNs or WPs via covalent disulfide bonds occurring during pasteurization of the cream [42], might have contributed to a more compact gel with reinforced interactions, increasing water retention within the yogurt gel for the BM-based yogurt [19]. In addition, SM treated at 300 MPa exhibited the lowest WHC value, which contrasts with previous studies that associated higher UHPH pressures to enhanced water retention due to increased interactions between WPs, CNs, and lipids [31,44,49]. However, these authors used whole milk rather than SM, and the homogenized fat globules are known to participate in increasing the strength of the gel network by participating as active filler particles, increasing the WHC [50,51].

In order to complete the characterization of set yogurts, firmness, which represents the gel network strength, was monitored through storage time. Statistical analysis showed that even though pressure treatment and time of storage did not impact firmness, it was significantly (*p* < 0.0001) influenced by the dairy source (BM or SM) used for yogurt production (Figure 3c). BM yogurts had a lower firmness than SM yogurts did, with average values of 70.34 N and 156.62 N, respectively (regardless of the time of storage and pressure level). These results agree with those of Le et al. (2011) who found that SM yogurts had a higher firmness than those fortified with 1, 2, 3, or 4% BMP [16]. However, very recently, Zhao, Feng, and Mao (2020) observed a higher firmness in yogurts fortified with 1−2% BMP than in a control SMP-yogurt [19], while 4% BMP yogurts had a lower firmness. In fact, exceeding a certain BM concentration might have adverse effects on the development of a stable yogurt gel, and it is assumed that PLs take more space and interact with the proteins, thereby disrupting the gel network [52]. Despite the fact that the pressure level did not significantly impact the firmness (*p* = 0.0901), we observed a decreasing tendency for both BM and SM yogurts. This tendency is in contrast with the results of yogurts obtained from milk treated between 100 and 300 MPa, which showed increased firmness with increasing pressure [31,44,53,54]. Globally, these studies show that while UHPH enhanced the interactions between WPs and CNs, in BM, the presence of smaller fat globules embedded in the protein network led to a higher firmness.

**Figure 4:**
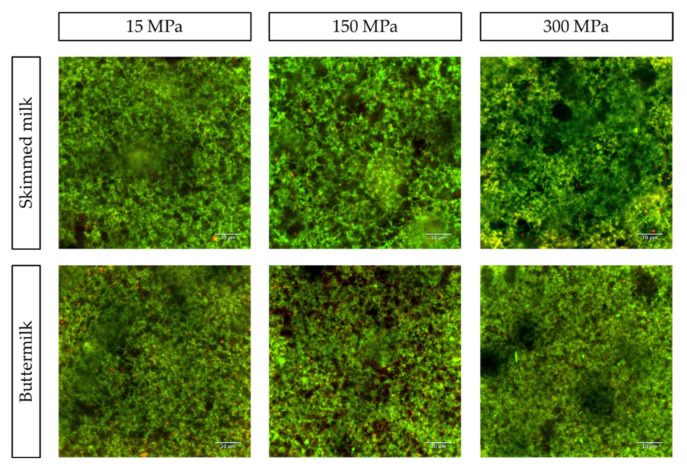
Confocal laser scanning microscopy (CLSM) images of set skimmed milk (SM) and buttermilk (BM) yogurts with different pressure applications (15, 150, and 300 MPa). Red color represents the phospholipids (PLs) labeled with Nile Red; green color represents the milk proteins labeled with Fast Green FCF. Scale bar (10 μm).

The final physico-chemical property evaluated was microstructure. The microstructure of set yogurts (SM and BM, each treated with 15, 150, and 300 MPa) was analyzed by CLSM and is shown in Figure 4, where the green color refers to proteins, and red to PLs. Overall, SM and BM yogurts exhibited different microstructures, which were impacted by UHPH treatments. The set yogurts made from SM exhibited protein clusters of larger size for all pressure treatments, whereas those made from BM had smaller protein particles, homogeneously distributed. The main difference in yogurts at 15 MPa was the PL content, which was, as expected, higher in BM than in SM. The few PLs present in SM seem to be of larger size than in BM, which might be due to the destructive effect of butter churning on the MFGM, resulting in smaller MFGM fragments in BM. In addition, PLs in BM yogurt were widely distributed throughout the gel matrix at 15 MPa. Increasing the pressure from 15 MPa to 150 MPa and 300 MPa largely changed the SM yogurt microstructure. As a matter of fact, we observed larger protein clusters forming large serum pores within the SM yogurt gels. However, for BM yogurts at 150 MPa and 300 MPa, the gel structures had very fine and continuous protein networks, which is supported by the decrease in particle size distribution of UHPH BM (unpublished data). In addition, for those pressures, PLs seemed to be distributed more heterogeneously and were bound to the protein network. The results for BM’s gel microstructure are in line with a previous study, which reported a dense structure with irregularly clustered protein aggregates for yogurt fortified with BMP due to a high content of proteins interacting with MFGM components [47]. Especially at 300 MPa, visible CN micelles can interact with MFGM fragments, trapping them within the yogurt protein network upon coagulation and preventing them from forming a more stranded gel [55]. These interactions between casein and MFGM would explain the lower firmness and viscosity observed for BM yogurts compared to the particulate gel observed for SM yogurts. The results of Le et al. (2011) support our observations of aggregated MFGM fragments within a homogeneous finely particulate casein network in BM yogurts [16]. The lower firmness observed for BM yogurts could be associated with the more homogeneous and finer gel observed and the occurrence of MFGM fragments within the gel, as indicated by Le et al. (2011) [16]. SM yogurts had stronger stranded networks, as can be seen from the higher contrast between the serum (black) and CN (green) phases. These microstructural changes in the protein gel network support the different physico-chemical and texture properties observed for both BM and SM set yogurts. For example, these differences could explain the enhanced WHC of BM yogurt as the homogeneously distributed PLs within the protein network increases water retention while reducing the firmness of the gel network [16].

### 3.3. Impact of UHPH Treatment on Physico-Chemical Properties of Stirred BM and SM Yogurt

**Figure 5:**
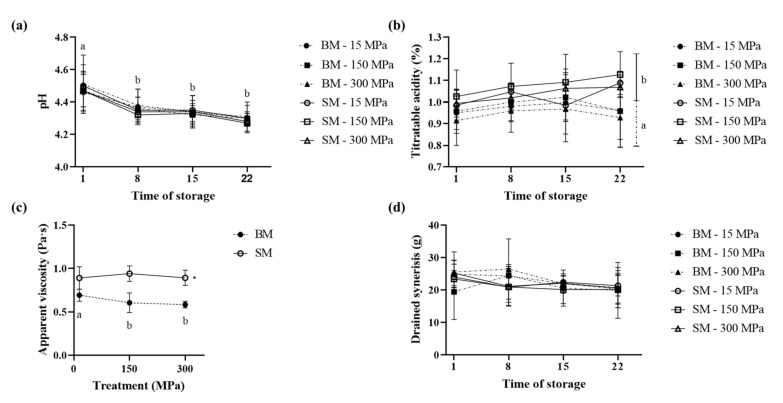
Evolution of physico-chemical properties ((**a**) pH, (**b**) titratable acidity, (**c**) apparent viscosity, and (**d**) drained syneresis) for stirred buttermilk (BM stippled line) and skimmed milk (SM. plain line) yogurts as a function of storage time (1, 8, 15, and 22 days) and pressure levels (15, 150, and 300 MPa).

Figure 5 represents the physico-chemical properties ((a) pH, (b) titratable acidity, (c) apparent viscosity, and (d) drained syneresis) of stirred yogurt as a function of the dairy source (BM and SM), homogenization pressure (15, 150, and 300 MPa), and storage time (days 1, 8, 15, and 22). No significant differences were observed in the pH of stirred yogurts (Figure 5a), between BM and SM dairy sources or between the different homogenization pressures. These results agree with those of Serra et al. (2008) who treated whole milk with pressures of 200 and 300 MPa prior to yogurt production [49]. In addition, an expected decrease in pH was observed upon storage at 4 °C, regardless of the source and pressure treatment. Indeed, the pH value decreased significantly (*p* < 0.0001) from 4.48 on day 1 to an average of 4.31 for days 8, 15, and 22. The drop in pH within the first week of yogurt storage agrees with results from a previous study by Moschopoulou et al. (2018), who noticed a drop in pH from day 1 (around pH 4.45) to day 7 (around pH 4.2) in semi-skimmed cow milk yogurt [43]. This decrease in pH was attributed to the residual lactose fermentation. However, Yildiz and Bakirci (2019) did not observe differences in pH in their BM- and WP-enriched yogurts with increasing storage time [17]. This can be explained by the presence of components such as WP, which are known to have great buffering capacity [56].

Interestingly, titratable acidity (Figure 5b), which is defined as the total acid concentration in a sample, was not impacted by storage time or pressure treatment. However, this property was influenced by the dairy source used for yogurt production. The titratable acidity of BM yogurt was significantly lower than that of SM yogurt (*p* = 0.0002), with averages of 1.01% and 1.06%, respectively. In contrast, another study, in which buffalo SM was replaced with 25, 50, 75, and 100% BM (fortified with 3% SMP), reported lower values for titratable acidity for the control SM yogurt (0.92%) compared to yogurt fortified with BM [18]. The authors demonstrated that a replacement with 100 and 75% BM (titratable acidity of 0.97%) led to even higher values for titratable acidity than 50% (0.95%) or 25% (0.93%). These differences between our study and the literature could be due to a slightly (however nonsignificant) higher protein content in SM yogurts (4.51%) compared to BM (4.28%), which might have induced the higher buffering capacity of SM, as observed by Trachoo and Mistry (1998) [57]. Indeed, the addition of ultrafiltered BM increased the protein content and, therefore, the buffering capacity followed by the titratable acidity of low-fat yogurt (titratable acidity: 1.39%) compared to the use of BMP (1.29%) [57].

The pressure, storage time, and dairy source parameters studied had different impacts on the physical properties of stirred yogurt. The apparent viscosity (Figure 5c) of stirred yogurts was affected by an interaction between pressure treatment and dairy source; however, no further differences were detected throughout the time of storage. This nonsignificance of the storage time contrasts with prior studies. Yildiz and Bakirci (2019), for example, observed irregular changes in apparent viscosity with storage time [17]. The interaction between homogenization pressure and dairy source was found to be significant (*p* = 0.015) with higher values for SM (0.94 Pa·s) than BM (0.63 Pa·s), whereas for SM yogurt, the apparent viscosity did not change with pressure; a slight but constant decrease was observed for BM yogurt from 15 MPa to 300 MPa. The higher values for SM compared to BM are in line with Yildiz and Bakirci (2019), who measured a lower viscosity for yogurts fortified with 2% BMP (+1% SMP) compared to those fortified with 3% SMP [17]. Recently, in contrast to our observations, Zhao, Feng, and Mao (2020) found that the viscosity of low-fat SMP-yogurt depends on the level of BMP fortification (0.5−4.0%) [19]. They found that viscosity increased significantly (up to ~60%) as the level of BMP incorporation increased to 2.0%, whereas the addition of 4% BMP resulted in a loss in viscosity (~13%). This suggests that the higher BM component content contributed to lowering yogurt viscosity, as observed in our study where BM was mainly used for yogurt manufacture. While the slightly higher protein content of the SM yogurt mix (Table 1) could have influenced the rheological properties of yogurts [58,59], the impact of BM addition seems to be related to increases in the amount of MFGM constituents. Studies have shown that while the fortification of small amounts of BMP (1−2%) increases viscosity, probably due to the emulsifying [48] and amphiphilic [47] properties of PLs and proteins, using higher amounts of BMP (4%) decreases viscosity [19]. The reinforced interactions between MFGM proteins and CNs or WPs via noncovalent or disulfide bonds [60], as well as interactions between PLs and WPs or β-CN, mainly via hydrophobic and electrostatic links (Gallier, 2012), probably contribute to the beneficial effect of MFGM fortification on physico-chemical yogurt properties, at least until a critical BMP concentration is reached. Consistent with our study, Le et al. (2011) concluded that BM supplementation contributes to the lower firmness of low-fat yogurts compared to the controls (12% SMP), which they also explained was due to the higher concentration of PLs from BMP [16]. Treatment of whole milk with UHPH has also been reported to induce interactions between denatured WP, lipids, and water, as well as interactions between CNs or between CNs and lipids, which enhances viscosity [61,62].

Finally, drained syneresis of the stirred yogurt was measured throughout the time of storage (Figure 5d). Drained syneresis measures the serum released due to shrinkage of the yogurt gel network [17], which is related to a textural defect of yogurts [63]. In our study, no difference was observed on the drained syneresis, regardless of the dairy source, pressure level, and storage time, with an average of 22.09 g of whey released. Our results are in contrast with those of Yildiz and Bakirci (2019) who found lower drained syneresis for control (3% SMP) yogurts compared to BMP-enriched yogurt [17].

**Figure 6:**
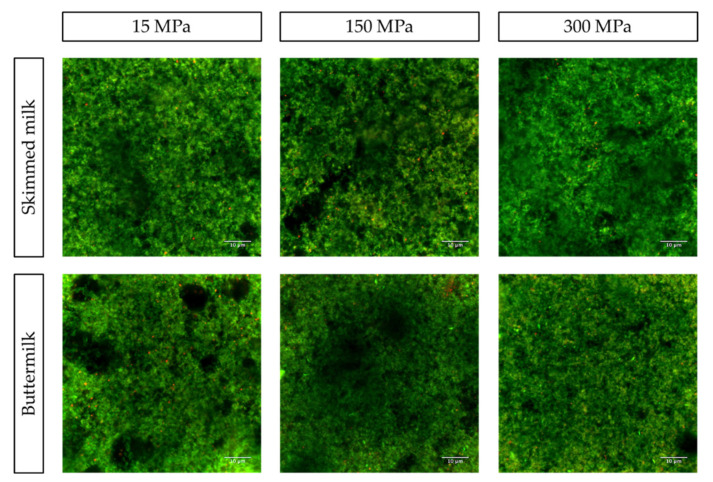
Confocal laser scanning microscopy (CLSM) images of stirred skimmed milk (SM) and buttermilk (BM) yogurts with different pressure applications (15, 150, and 300 MPa). The red color represents the phospholipids (PLs) labeled with Nile Red; the green color represents the milk proteins labeled with Fast Green FCF. Scale bar (10 μm).

Just as for set yogurts, the microstructure of stirred yogurts (SM and BM, each treated with 15, 150, and 300 MPa) was also studied using CLSM (Figure 6). Again, different microstructures were observed for stirred SM and BM yogurts subjected to different pressure treatments. For SM yogurts, changes within the microstructure with increasing pressure seem to be less distinct than for BM yogurts. At all three pressures, PLs were evenly distributed throughout the gel. However, at 150 MPa, more serum pores of larger size were observed than at 15 and 300 MPa. In addition, SM yogurts pretreated with 300 MPa had a more homogeneous gel with smaller protein particles than those pretreated with 15 MPa. In BM yogurts, however, more PLs were present in the gel, which exhibited finer and more homogeneously distributed protein aggregates, the difference being particularly visible at 300 MPa. At 15 MPa, BM gels contained large serum phases, which lessened with increasing pressure. Indeed, at 300 MPa, serum phases were virtually absent, whereas SM yogurt still exhibited larger serum pores at 300 MPa. A very fine gel with small protein particles was observed for BM 300 MPa. Further, PL particles were bound to protein particles and were of smaller size compared to those in gels treated with 15 and 150 MPa. This might be due to the effect of pressure-induced particle size reduction on proteins and PLs, as previously explained. The higher apparent viscosity of SM over BM stirred yogurts can probably be traced back to the stronger gel network of SM compared to BM set yogurts. For BM yogurts, the decrease in apparent viscosity with increasing pressure could be attributed to the wider distribution of smaller PLs at 150 and 300 MPa, possibly impacting protein gel strength in a way that decreases apparent viscosity. As observed for set yogurts, the interaction between PLs and the protein network at higher pressures (150 and 300 MPa), combined with the more denatured and aggregated protein in BM, might have impaired the formation of a stable gel [55].

## 4. Conclusions

This work studied the impact of UHPH on BM to improve its techno-functionality for incorporation into yogurt applications. The results showed that UHPH treatment more drastically impacted BM proteins, which underwent more denaturation and aggregation than SM proteins did, thus impacting the physico-chemical and textural properties of the set and stirred yogurts produced from BM and SM. In addition, the gel microstructure was influenced by the UHPH-treatment and depended on the dairy source. Indeed, at the highest pressure (300 MPa), set SM yogurts presented large protein clusters with large serum pores, while set BM yogurts produced finer and more homogeneously distributed protein particles that interacted with PL and correlated with lower firmness. This work represents the first step in understanding the impact of UHPH on BM for the production of yogurt. It can support the development of new technology for the valorization of BM in order to take advantage of the beneficial effects of MFGM components on human health. Future research could focus on enriching BM with cream prior to UHPH treatment to produce a full-fat yogurt and investigating the impact of lipids on UHPH-treated yogurt properties. It could also focus on the interaction effect between lipids and proteins by studying their impact on the gel network and yogurt microstructure.

## Figures and Tables

**Table 1:** Composition of the standardized buttermilk (BM) and skimmed milk (SM) mix used for yogurt production.

	Buttermilk	Skimmed Milk
Total solids (%)	11.88 ± 0.03 *	11.89 ± 0.11
Lipids (%)	0.59 ± 0.07	0.13 ± 0.05
Proteins (%)	4.28 ± 0.06	4.51± 0.08
Lactose (%)	6.26 ± 0.05	6.49 ± 0.11

* Mean values (*n* = 4) ± standard deviation.

## Data Availability

Not applicable.
